# Echocardiographic evaluation in Dorper ovine fetuses: Applications and limitations

**DOI:** 10.1002/vms3.384

**Published:** 2020-10-29

**Authors:** Amanda Sarita Cruz‐Aleixo, Mayra de Castro Ferreira Lima, Ana Luísa Holanda de Albuquerque, Raphael Tortorelli Teixeira, Renata Alves de Paula, Marina Cecília Grandi, Danilo Otávio Laurenti Ferreira, Miriam Harumi Tsunemi, Simone Biagio Chiacchio, Maria Lucia Gomes Lourenço

**Affiliations:** ^1^ School of Veterinary Medicine and Animal Science São Paulo State University (Unesp) Botucatu São Paulo Brazil; ^2^ Diplomate in Veterinary Medicine Lençóis Paulista São Paulo Brazil; ^3^ Coordination of Integral Technical Assistance Office of Development of Bauru Secretary Agriculture of the State of São Paulo Botucatu Brazil; ^4^ Institute of Biosciences São Paulo State University (Unesp) Botucatu São Paulo Brazil

**Keywords:** development, fetal monitoring, heart, pregnancy, sheep, ultrasound

## Abstract

In this study we aim to show the application of ultrasound evaluation of the fetal heart in the ovine species, as well as its limitations in the field. Ten Dorper sheep, without any sedation, were evaluated starting from the second month of pregnancy through transabdominal ultrasound with an ultrasound device equipped with a convex transducer. Images of the fetal heart were obtained through maternal abdominal ultrasound by identifying the position of the fetus and conducting the following measurements: length and diameter of the heart, dimensions of the right and left ventricles and dimensions of the right and left atria. The measurements could only be conducted with acceptable precision starting from the third month of pregnancy. There was a significant difference only for left ventricle diameter, which was larger in the fifth month of pregnancy. The echocardiographic evaluation of the fetus enables monitoring the heart development identifying early fetal viability, assessing inadequate events that could put the pregnancy at risk, especially for production animals. For the experimental design of research employing production animals, it is important to consider, among other factors, the limitations of the evaluation on the field, such as restraining the animals, the stress caused by handling and environmental conditions, temperature, luminosity, facilities available and the qualifications of the team.

## Graphical abstract

Fetal ultrasound in sheep is an examination that can help identify fetal viability by analysing cardiac dimensions in sheep. There are limitations of the technique to the field. The method may contribute to the reduction in losses during pregnancy.

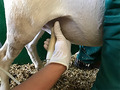

## 1 INTRODUCTION

Ultrasound monitoring of the fetal heart is extremely important to detect congenital anomalies (Kovalchin, 2004; Suguna et al., 2008). Transabdominal and rectal ultrasonography are used to detect twins, document fetal position, estimate fetal size, evaluate placental integrity and fetal fluid clarity, and gain a general impression of fetal well‐being (Le Blanc, 1996).

In humans and animals, fetal heart rate pattern, level of activity and degree of muscle tone are sensitive to hypoxaemia and acidaemia. Techniques such as cardiotocography, ultrasonography and maternal perception of fetal movement can identify fetuses that are either suboptimally oxygenated or acidaemic because of placental dysfunction (Vinzce et al., 2019). In human medicine, biometric alterations in the structure of the heart are assessed using fetal size parameters when there are suspicions of congenital cardiopathies or growth alterations in the heart unrelated to cardiac diseases (Giannico et al., 2016; Kulkarni, 2013), and fetal echocardiography has become the standard prenatal diagnostic test for fetuses with suspicions of heart anomalies (Smrcek et al., 2006). During pregnancy, the heart of the fetus undergoes several modifications until the formation of the blood vessels, when it becomes the central organ responsible for pumping the blood through rhythmic contractions (Saul et al., 2014).

Detecting pregnancy early in sheep is of utmost importance for identifying animals that are sterile or unsuitable for reproduction for slaughter. However, it is difficult to differentiate between failures to return to oestrus due to the occurrence of seasonal anoestrus at the end of the reproductive period (Garcia et al., 1993). The diagnosis of pregnancy in sheep involves an economic component for the producers and, when done early, is useful to ensure the adequate nutritional handling of the animal and to reduce the risk of toxaemia during pregnancy (Aiumlamai et al., 1992; Chalhoub et al., 2001; Fridlund et al., 2013).

This study aimed at, after the diagnosis of pregnancy, assessing the cardiac development in ovine fetuses during pregnancy considering that the species is often used in experimental studies in human neonatology due to the similarities of the hearts of both species. Identifying the size of the heart and possible congenital cardiopathies may contribute towards further research about fetal surgical techniques. The study also aimed at discussing the technical limitations of the ultrasound technique in the field.

## 2 MATERIALS AND METHODS

### 2.1 Animals

Ten Dorper sheep (*Ovisaries*) were evaluated starting from the second month of gestation. All sheep underwent physical examination and were excluded if any clinical abnormalities or extremes of body condition score were detected. All sheep were vaccinated and dewormed. The average age of the females was 4.4 ± 1.35 years (minimum 2 years and maximum 7 years).

The ewes were kept in a semi‐finishing system, receiving feed twice a day. The composition of the mixture was 65% ground corn, 10% wheat bran, 20% soybean meal and 5% nucleous sheep feed concentrate (NSFC). The basic composition of the NSFC is illustrated in the Table 1.

**TABLE 1:** The basic composition of the nucleous sheep feed concentration

Mineral	Amount
Calcium (minimum)	120.0 g/kg
Calcium (maximum)	220 g/kg
Phosphorus (minimum)	18.00 g/kg
Sodium (minimum)	78.00 g/kg
Magnesium (minimum)	7700.00 mg/kg
Potassium (minimum)	10.00 g/kg
Iron (minimum)	250.00 mg/kg
Zinc (minimum)	1875.00 mg/kg
Manganese (minimum)	650.00 mg/kg
Iodine (minimum)	30 mg/kg
Selenium (minimum)	8.00 mg/kg
Cobalt (minimum)	20.00 mg/ kg
Monesine	600.00 mg/kg

The study was conducted according to the animal welfare guidelines and authorized by the owner, who consented to the experimental plan and the procedures conducted.

The study was conducted on a property located at the city of Botucatu, SP, Brazil, on the Rubiao Junior district, latitude S‐22.902107 and longitude W‐48.516534, from July 2017 to December 2017. Ten non‐pregnant sheep assessed by ultrasound prior to the study and submitted them to the oestrus‐inducing protocol for fixed‐time artificial insemination (FTAI).

### 2.2 Fetal ultrasound evaluation

The first data from the fetuses were collected starting at 42 days after the FTAI protocol, as it is recommended that animals are not to be handled for a period of at least 28 days after the protocol to avoid losing embryos due to excessive handling. For the ultrasound evaluation of the fetuses, the study employed a portable ultrasound device My LabTM30 Vet Gold (Esaote®‐Esaote Healthcare do Brasil, São Paulo‐SP, Brazil) equipped with a convex 5.0 MHz transducer at a frequency of 3.0 MHz. Imaging exams were always performed by the same operator (ASCA).

**FIGURE 1:**
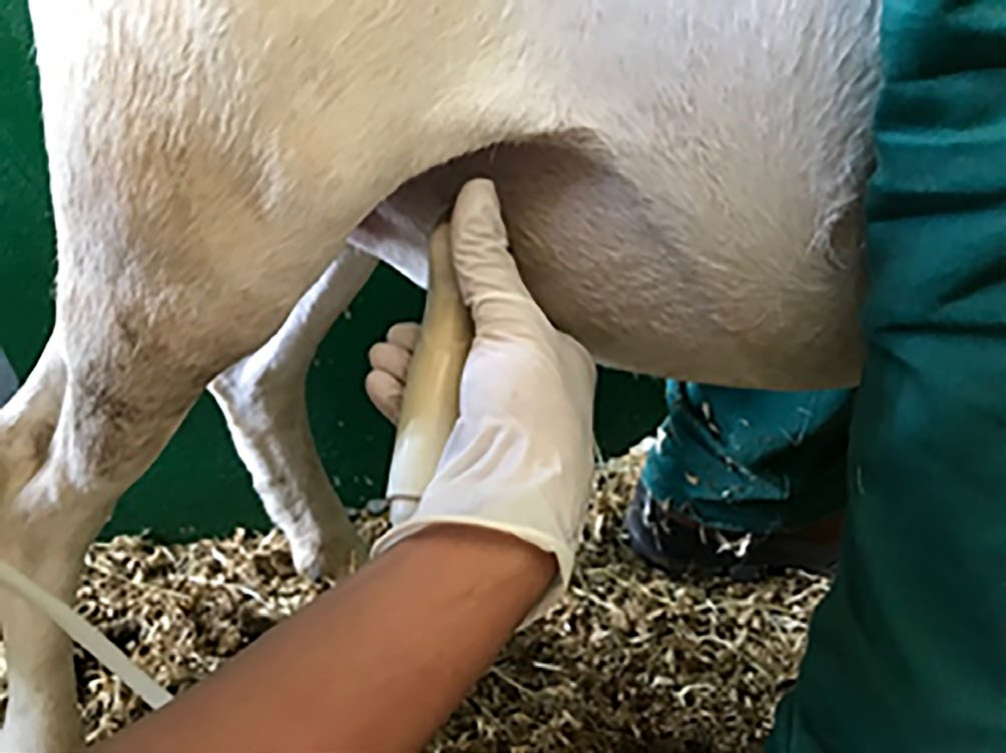
Position of the operator and the demonstration of inguinal region for fetal identification in Dorper sheep

To acquire the images, the abdominal region of the ewe was properly epilated and cleaned. Transabdominal ultrasonography was performed with the sheep in a stationary position through manual physical restraint (manual), with the operator positioned laterally to the animal. The transducer was placed at the region of the right caudal abdominal quadrant, near the inguinal region (Figure 1), initially to identify the fetus, but due to the movement of the fetus the transducer was repositioned to identify the cardiac region. Therefore, the transducer was positioned to obtain longitudinal sections of the fetus and subtle rotational adjustments were made until images similar to those obtained through conventional echocardiography were obtained.

**FIGURE 2:**
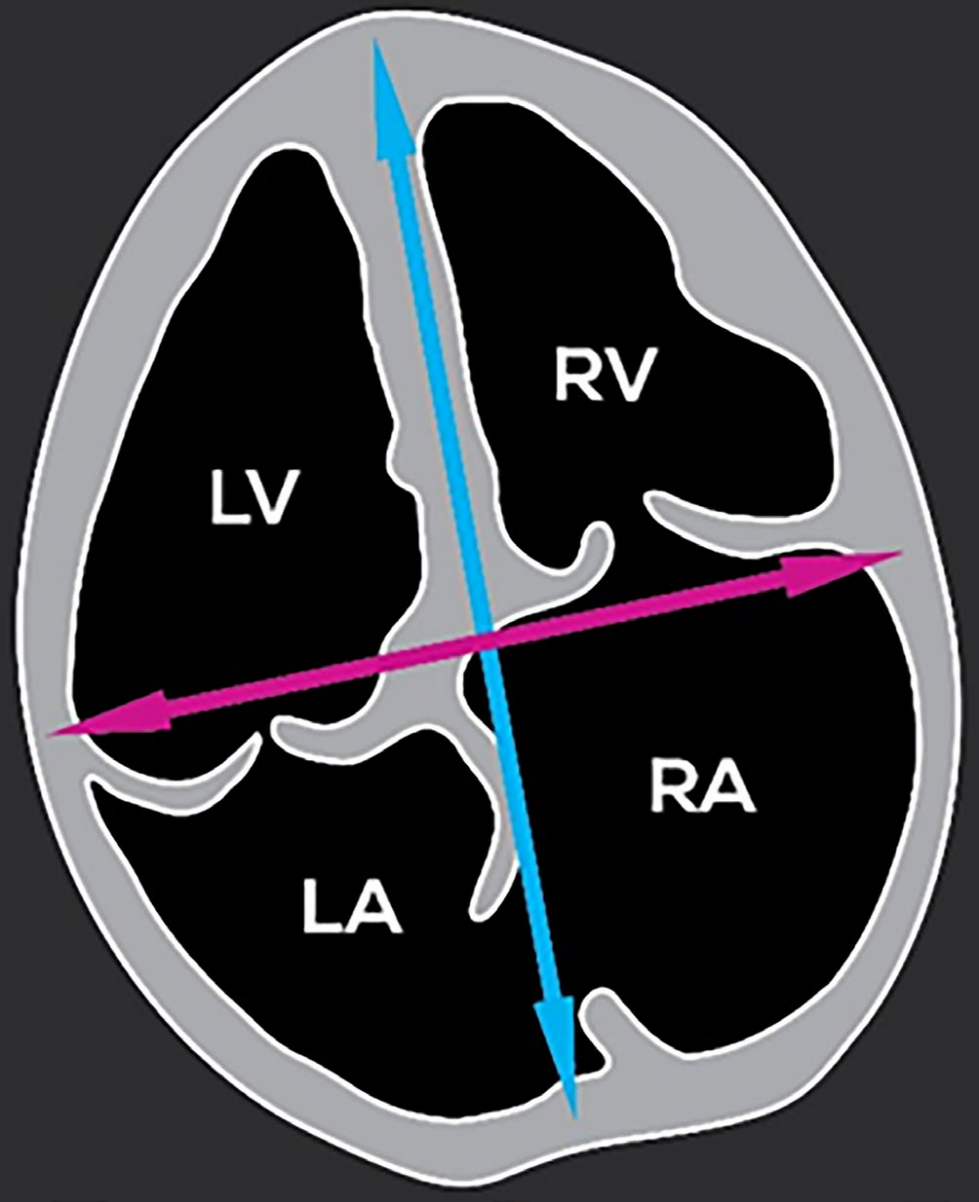
Schematic representation of a longitudinal section of the four cardiac chambers showing the length and diameter measurements of the fetal heart

**FIGURE 3:**
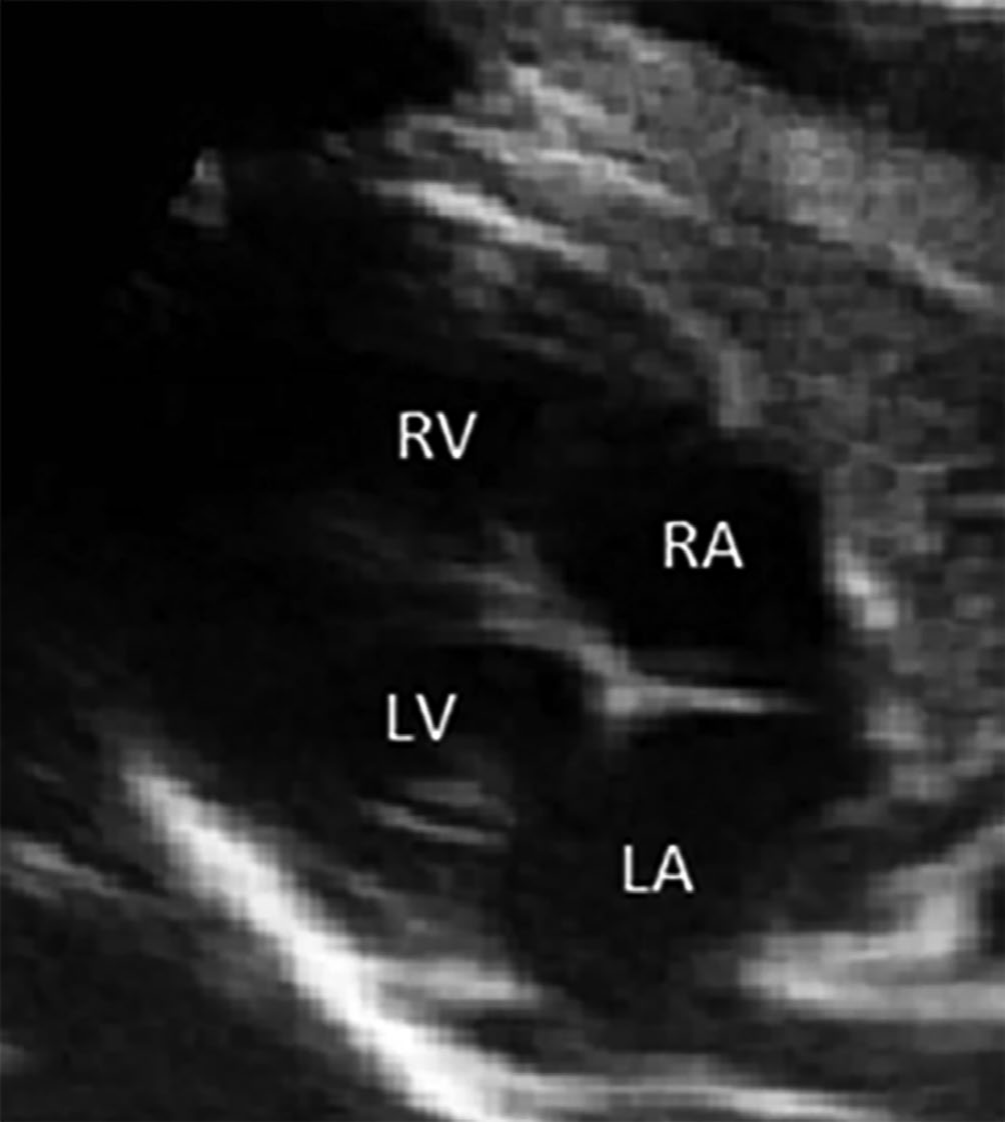
Fetal echocardiogram (4th month) in an ovine. Longitudinal section on the long axis showing the four cardiac chambers of the fetus

**FIGURE 4:**
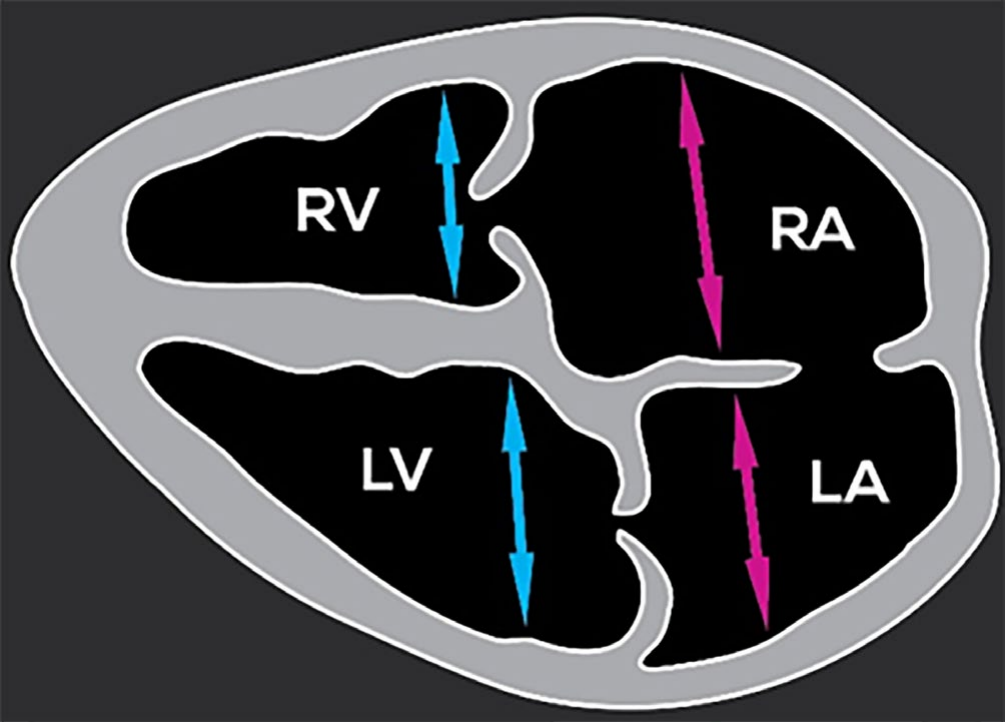
Schematic representation of a longitudinal section showing the measurements of the atrial and ventricular chambers

**FIGURE 5:**
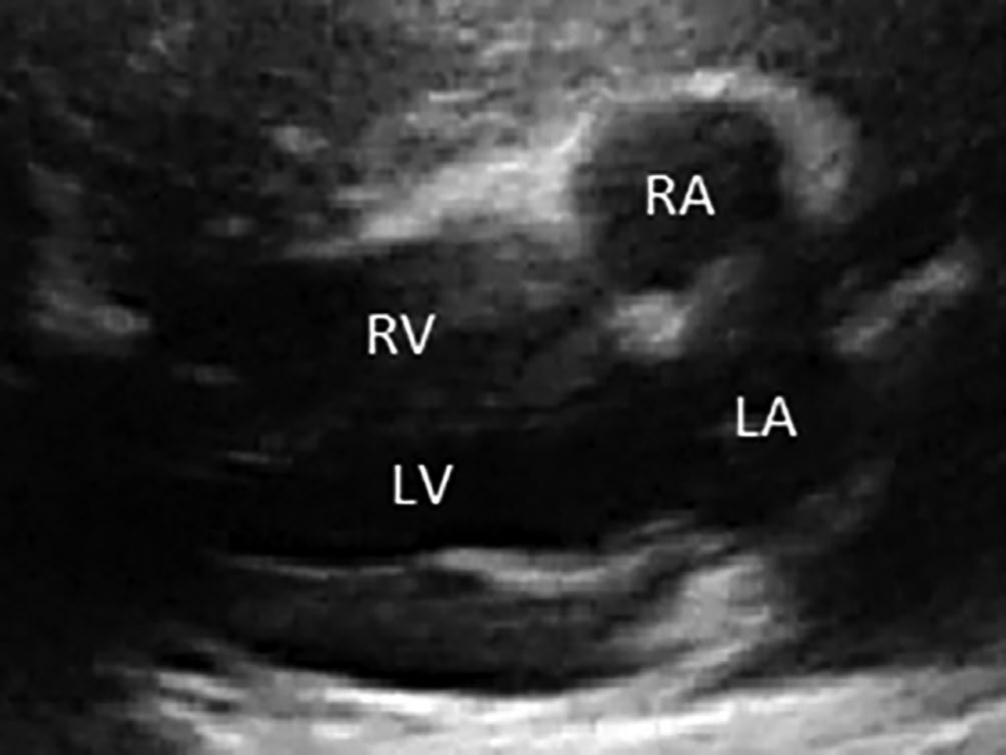
Fetal echocardiogram (4th month) in an ovine. Longitudinal section on the long axis showing the ventricular and atrial chambers

For each fetus, after identifying the cardiac chambers, the cardiac parameters were measured, if possible, using bidimensional fetal echocardiography through transabdominal ultrasound of the pregnant sheep. The parameters measured were length and diameter of the heart, dimensions of the right and left ventricles and dimensions of the right and left atria (Figures 2, 3, 4 and 5). When the fetuses were twins, the measurements were taken only from one fetus and the measurements were not conducted when the position of the fetus was unsuitable for measurements.

The cardiac chambers were measured at their largest diameter, at the end of diastole. The atrial measurements were taken from the atrial walls and the ventricular measurements from the segments where the valves were closed, excluding the interventricular septum. Measurements were taken preferentially with the fetus in a longitudinal position, longitudinal axis four chambers, with the spine of the fetus parallel to the maternal spine. Measurements were not taken with the fetuses in transverse or oblique positions.

### 2.3 Statistical analysis

The results are shown as median, minimum and maximum values, interquartile ranges. The normality test employed to analyse the parameters was the Kolmogorov‐Smirnov test; comparison between the proposed moments was conducted employing Friedmann's test because all measures were not normally distributed. The significance level considered for the study was 5%.

## 3 RESULTS

Of the ten females assessed in the study, eight were pregnant with a single fetus and two were pregnant with twins. Table 2 shows the echocardiographic parameters of the fetuses obtained in this study, with the measurements being feasible starting from the third month of pregnancy due to the size of the heart.

**TABLE 2:** Fetal echocardiographic evaluation (diastole) (median, minimum, maximum, quartile intervals) in Dorperovines

Measurement (cm)	3rd month	1st quartile	3rd quartile	4th month	1st quartile	3rd quartile	5th month	1st quartile	3rd quartile	*p*
Long axis	3.34 (2.66;3.79)	2.81	3.70	3.37 (3.28;6.35)	3.31	3.94	4.16 (2.97;6.35)	3.18	5.53	.411
Heart diameter	2.38 (2.11;3.07)	2.22	2.86	2.42 (2.34;3.10)	2.38	3.02	2.97 (2.38;3.75)	2.52	3.44	.241
RA Diameter	0.93 (0.44;1.70)	0.61	1.44	1.00 (0.75;1.42)	0.84	1.32	1.26 (0.81;1.70)	1.00	1.65	.204
LA Diameter	0.73 (0.51;1.15)	0.63	0.98	0.80 (0.61;1.42)	0.64	1.15	1.03 (0.85;1.42)	0.86	1.23	.211
RV Diameter	0.74 (0.41;1.16)	0.51	0.99	0.91 (0.63;1.27)	0.70	1.25	1.16 (0.72;1.53)	0.77	1.27	.623
LV Diameter	0.57 (0.44;0.75)	0.47	0.73	0.68 (0.54;0.98)	0.56	0.80	0.77 (0.63;1.07)	0.63	1.03	*.030

Note

Kolmogorov‐Smirnov normality test; comparison between moments, Friedmann's test; interquartile intervals for multiple comparisons; *significance: *p* < .05; RA: right atrium; LA: left atrium; RV: right ventricle; LV: left ventricle.

These results show that the largest dimensions for each echocardiographic variable occurred at the 5 months of pregnancy. The measured parameters do not present statistically significant differences between each time point, except for the diameter of the left ventricle (LV). Despite the absence of significant difference between the evaluated periods, we observed that the LV diameter and the cardiac length, as well as the dimensions of the atrial and ventricular chambers accompanied the fetal development being larger in the last gestational month.

Through the analyses performed, no structural changes compatible with congenital heart disease were observed and the viability of all fetuses was maintained throughout the gestational period evaluated.

## 4 DISCUSSION

The fetal evaluations started at the second month of pregnancy in this study, but at that time it was not possible to measure the cardiac chambers due to the small size of the heart and a worsened visualization caused by interference from other tissue and artefacts when acquiring the images, including the maternal adipose tissue, excessive fetal movement and the maternal gastrointestinal tract, which is something that also happens in humans, who suffer interference from high maternal body mass scores and the depth of the fetus (Gardiner, 2001; Tatani, 1997).

Starting at the third month of pregnancy, it was possible to assess the fetal heart, but the quality of the images was higher in the last month of pregnancy, which is in line with the cardiac evaluation in human fetuses. In humans, the beats of the fetal heart can be viewed by bidimensional echocardiography starting at the 6th week of pregnancy (Carvalho et al., 2004), but an adequate structural analysis is only possible starting at the 16th week. During this period, the fetal heart is still very small and a complete study is often not possible (Gardiner, 2001).

This study observed artefacts when acquiring the images, caused mostly by the maternal adipose tissue layer, tissue overlap of fetuses when there were twins and acoustic shadows created by the contents of the maternal gastrointestinal tract. In humans, better ultrasonographic windows are present for fetal evaluation, but artefacts causing shadows in the cardiac region also happen, mostly due to maternal obesity. The ideal timespan to observe the fetal heart in humans goes from the 18th to the 24th week of pregnancy, when the fetus is covered in a large volume of amniotic liquid (Lopes, 2016; Wiley, 2006). During the third trimester, the spine of the fetus is often anterior and the ribs are more calcified, creating shadows in the cardiac area, thus, making accurate cardiac assessment difficult at this stage (Allan, 2003).

In this study, the echocardiographic evaluation of the fetus during the second month of pregnancy observed that the dimensions of the heart were relatively small, but as the pregnancy progressed, we noted that, despite the larger measurements of the heart, there were still artefacts. We believe that this may be explained by the movement of the fetus, which was exacerbated by maternal agitation due to handling. In addition, the breed selected may have contributed due to the abundant layers of adipose tissue.

We also consider important to note the technical limitations that happen when conducting imaging examination on the field. Both the experimental design and the behaviour of the animal should be considered when acquiring ultrasound images. The animals included in the study were restless during handling, which translated into more fetal movement as the restlessness of the mothers increased, which represented a limiting factor to acquiring diagnostic images. In addition, some images were acquired closer to the inguinal region, which caused discomfort to the ewes.

Vincze et al. (2019) conducted a study to evaluate equine fetal parameters using transabdominal ultrasonography by compiling six factors: fetal heart rate, fetal aortic diameter, maximal fetal fluid depth, uteroplacental contact, uteroplacental thickness and fetal activity. The authors concluded that equine fetal tests do not reach the high accuracy of human surveillance tests but are promising tools for later development on large patient groups.

The Technical Bulletin for cardiac ultrasound examinations in human fetuses issued by the AIUM (American Institute of Ultrasound in Medicine) (2013) recommends that fetal echocardiographic examinations should be conducted between the 18th and 22nd weeks of pregnancy. This is when the quality of the images is ideal, resulting in more precise diagnoses, but even at 18 weeks, the fetal heart is a small structure (Drose, 2001) and even at later stages of the pregnancy, the echocardiographic evaluation may be compromised by an attenuation caused by the cranium, ribs, spine or limbs of the fetus, or by the reduction in the levels of amniotic liquid as the pregnancy progresses (Lopes, 2016).

In our study, as is the case in humans, the dimensions of the RV and LV were very similar and within a centimeter, which highlights the similarities between the hearts of both species and justifies the use of ovines as experimental models for cardiovascular research. In humans, the cardiac cavities triple or quadruple their size from the 17th week until the end of the pregnancy, revealing a linear growth pattern regarding age and diameter (Yagel et al., 2007). The ratio of the dimensions of the LV and the RV was around one and remained constant throughout the pregnancy. The measurements of the ventricles are similar, but we have to consider that the geometries of the right and left ventricles are different (Rudiski et al., 2010), as are the morphologies of the trabeculae and ventricular exits (Rublin et al., 1977).

Translational studies are developed to better reproduce human physiological conditions and the main advantage of animal trials is that the controls may be selected according to their suitability rather than to what is permissible in a patient (Swanson & David, 2015). The experimental environment is an important factor, particularly in sheep, which are animals that do not adapt favourably to handling and alter their behaviour. Different imaging techniques should be considered according to the situation; in this study, with the sheep kept stationary, we chose the transabdominal technique in lieu of the transrectal technique, but dorsal decubitus may represent an alternative to make acquiring the images, as containing the animal is easier in this position.

The transrectal examination is more efficient than the transabdominal method until the 35th day of pregnancy. Between the 35th and 70th days of pregnancy, both methods appear to be equally efficient. During the second half of the pregnancy, the transabdominal technique allows better accuracy due to the position of the intrapelvic uterus, located above the bladder and the horns deviate in a ventrolateral direction. In the final third of the pregnancy, due to the development of the fetal membranes and the accumulation of fluids, the uterus deviates in a cranioventral direction inside the abdomen (Renaudin et al., 1997). Therefore, we chose the transabdominal technique for this study also due to the changes in the position of the uterus that happen as the pregnancy progresses.

Other factors to be considered for fetal cardiac evaluation of the ovine field are the operator's experience, as the ideal is that the tests be performed in a short time to avoid animal stress; the number of fetuses and the breed are also relevant in this context, as, in the Dorper breed, the evident adipose tissue promotes artefacts in the acquisition of the image and the animals of the present study were in a corporal score of 4.5, being overweight due to overfeeding. Reef et al. (1996) states that time of examination is critical for evaluation of equine fetuses by ultrasound technique because horses usually get bored within a few minutes and are not very co‐operative without sedation. The use of sedatives should be minimized or avoided in pregnant animals. The faster the examination, the more time can be saved for the diagnosis. According to the authors, in equine hospitals, several transducers are available with trained personnel, but under field conditions this is hardly achievable, evidencing that the ultrasound evaluation in the field has its restrictions.

The present study only evaluated fetal cardiac measurements. We did not employ the Doppler method for HR assessment which could have contributed to the assessment of fetal viability. However, qualitative analysis allows the identification of cardiac chambers, and heart valves may assist in the suspicion of congenital heart disease when cardiac size does not follow development or when cardiac chambers and valves are identified with inadequate positioning. The ultrasound method is widely used for pregnancy diagnosis in the agricultural sector. As it is already used in reproductive practices, extending analysis for fetal heart evaluation, even through qualitative analysis, can optimize pregnancy management, where detection of cardiac structural abnormalities or reduced movement of heart chambers may allow intervention in pregnancy, reducing losses and/ or costs with unviable animals.

Therefore, fetal evaluation is important to identify potential risks to the fetuses and allow them to benefit from early interventions, avoiding adverse effects that could potentially lead to death, particularly in high‐risk pregnancies. According to Nomura et al. (2009), one of the adaptative responses of the fetus to hypoxemia is a hemodynamic compensation mechanism involving the stimulation of the autonomic nervous system of the fetus, resulting in increased peripheral vascular resistance and heart rate in the fetus. In cases of placental alterations and progressive fetal hypoxemia, abnormalities in the fetal heart rate are considered early signs of a compromised fetus. Reductions in fetal movement may be associated with congenital abnormalities, which are also something that may be identified by maternal transabdominal ultrasound, highlighting the importance of the method for intrauterine life.

Some limitations are attributed to the present study. The relatively small sample size may have contributed to no significant differences in cardiac dimensions over time and a larger animal sample could have more clearly explained cardiac development. In the analysis of twin fetuses, it was not possible to ensure that the measurement of the cardiac size of the same fetus would be performed at all times of collection. We did not use the Doppler method to assess fetal heartbeat, which could have contributed to our assessment of HR, overall heart health and fetal viability. Post‐mortem cardiac size assessment could more effectively represent heart size and development, but was not performed in the present study.

## 5 CONCLUSIONS

The dimensions of the heart follow the development of the fetus and sheep dimensions are similar to the observed in humans. This is important for monitoring pregnancy for fetal viability, as in the absence of fetal viability, pregnancy can be ruled out, resulting in negative economic impacts on producers, especially in inseminated animals, where investments for good quality semen acquisition as well as pregnancy maintenance are high and its return depends on the success rate of animals with pregnancy positive insemination. However, the experimental environment, operator experience, the number of fetuses and the breeds chosen are factors that should be considered when acquiring ultrasound images. Fetal echocardiography is effective and useful for normal cardiac morphological evaluation in sheep animals and is extremely important to identify fetuses in risk and allow short‐term interventions.

## CONFLICTS OF INTEREST

The authors declare no conflicts of interest.

## AUTHOR CONTRIBUTION

**Amanda Sarita Sarita Cruz Aleixo:** Formal analysis; Writing‐original draft. **Mayra de Castro Ferreira Lima:** Data curation; Formal analysis. **Ana Luísa Holanda de Albuquerque:** Data curation; Software. **Raphael Tortorelli Teixeira:** Resources; Visualization. **Renata Alves de Paula:** Conceptualization; Investigation. **Marina Cecília Grandi:** Methodology; Supervision; Visualization. **Danilo Otávio Laurenti Ferreira:** Project administration; Writing‐review & editing. **Miriam Harumi Tsunemi:** Formal analysis. **Simone Biagio Chiacchio:** Funding acquisition; Methodology; Supervision. **Maria Lucia Gomes Lourenço:** Funding acquisition; Project administration; Writing‐review & editing.

### PEER REVIEW

The peer review history for this article is available at https://publons.com/publon/10.1002/vms3.384.
